# Changes in anxiety and depression among public health workers during the COVID-19 pandemic response

**DOI:** 10.1007/s00420-023-02002-6

**Published:** 2023-07-20

**Authors:** Kahler W. Stone, Meredith A. Jagger, Jennifer A. Horney, Kristina W. Kintziger

**Affiliations:** 1 Department of Health and Human Performance, Middle Tennessee State University, Murfreesboro, TN 37132 USA; 2 Austin, TX 78746 USA; 3 Epidemiology Program, University of Delaware, 100 Discovery Blvd, Room 731, Newark, DE 19713 USA; 4 Department of Environmental, Agricultural, and Occupational Health, College of Public Health, University of Nebraska Medical Center, Omaha, NE 68198 USA

**Keywords:** COVID-19, Anxiety, Depression, Public health, Workforce, Longitudinal

## Abstract

**Objectives:**

The COVID-19 pandemic has negatively impacted mental health indicators, leading to an increase in symptoms of anxiety and depression in both the general population of adults and children and many occupational groups. This study aims to examine changes in anxiety and depression among a cohort of public health workers in the U.S. during the first year of the COVID-19 pandemic and identify potential risk factors.

**Methods:**

Longitudinal data were collected from a sub-sample (*N* = 85) of public health workers in 23 U.S. states who completed two surveys in 2020 and 2021. Information on background characteristics, personal well-being, and work environment as well as validated scales to assess generalized anxiety disorder (GAD), depressive disorder, and burnout was collected. Data were analyzed using Stata Version 17, and significant differences were determined using Pearson’s Chi^2^ and Fisher’s Exact tests.

**Results:**

The proportion of those reporting GAD (46.3% to 23.2%) or depression (37.8% to 26.8%) improved from Survey 1 to Survey 2 overall; symptoms of anxiety saw the largest improvement. Persistent depression was associated with sustained burnout, changes in social support, and days worked per week.

**Conclusion:**

Public health workers experienced elevated levels of anxiety and depression during the initial pandemic response, but a reduction in these symptoms was observed in the subsequent year after vaccines had become widely available. However, unmet needs remain for ongoing workplace mental health supports to address burnout, as well as for additional emotional supports outside of work for public health professionals.

## Introduction

The COVID-19 pandemic has had a significant negative impact on mental health indicators in both the general population and healthcare workers in the United States and globally (Santarone et al. 2020; Serafini et al. 2020). During the COVID-19 pandemic response, healthcare workers were at increased risk of depression due to added stressors, such as longer work hours, increased workload, and fear of contracting and spreading the virus. In the first year of the pandemic, the prevalence of symptoms of anxiety and depressive disorders has increased by three to four times the pre-pandemic levels (Czeisler 2020; National Center for Health Statistics 2020; Wright et al. 2021).

The pandemic response also placed a considerable mental health burden on the public health workforce, specifically with elevated rates of anxiety and depression reported (Bryant-Genevier 2021; Pfender et al. 2022; Stone et al. 2021). Most studies on the mental health of the public health and healthcare workforces have focused on those with patient-facing occupations (e.g., doctors and nurses). Several systematic reviews and meta-analyses have been conducted to estimate anxiety and depression prevalence in healthcare workers and report wide ranges of prevalence for anxiety (12–68%) and depression (9–65%) (Chirico et al. 2021; Luceño-Moreno et al. 2022; Uphoff et al. 2021; Vizheh et al. 2020). In addition to cross-sectional studies, several longitudinal studies on healthcare workers have been conducted to track their mental and physical health (Conceição et al. 2021; López Steinmetz et al. 2022; Luceño-Moreno et al. 2022; Sasaki et al. 2021; Van Steenkiste et al. 2022; Wilson et al. 2022).

Some longitudinal data are also available on the health impacts of the pandemic response on disaster responders and rescue workers more generally. For example, in a literature review, Mao et al. (2018) reported high risks of psychological problems among rescue workers, including firefighters, police officers, military personnel, healthcare workers, and volunteers, for up to 10 years after a disaster deployment (Mao et al. 2018). In a systematic review, Brooks et al. (2016) found limitations in longitudinal studies reporting psychological distress among disaster responders, including unexplained loss to follow up and a lack of standard definitions and terminology or comparable outcome measures (Brooks et al. 2016). These limitations may be why a meta-analysis of studies investigating the role of social support on first responder mental health found no differences between the effect of social support in cross-sectional versus longitudinal studies (Prati and Pietrantoni 2010).

A longitudinal study focusing on the impact of the pandemic among healthcare workers suggests that both social and professional supports mitigated the impacts of the COVID-19 pandemic response on healthcare worker moral injury, burnout, and psychiatric distress over a three month period of the initial COVID-19 surge in the U.S. (March 2020–July 2020) (Hines et al. 2021). Emergency medicine resident physicians in three U.S. states surveyed in March 2020 and May 2020, reported that stress levels actually decreased over time as testing and personal protective equipment became more accessible for healthcare workers (Baumann et al. 2021). An exception to this was among female respondents, who had an increased risk of post-traumatic stress disorder (PTSD) as measured on a primary care PTSD screening scale (Baumann et al. 2021). Another short-term longitudinal study (i.e., 2 months) focusing on nurses working in frontline healthcare in Europe between April and June of 2020 also showed declining trends in depression, anxiety, and somatization; however, distress scores remained high at both time points, and 10% of respondent’s scores were predictive of PTSD (Van Steenkiste et al. 2022).

Much of the research about the health impacts of disasters and public health emergencies are case studies that characterize the impacts of a single type of disaster in a specific geographic location. Comparative data using common measurements over time and across locations are needed to understand the longitudinal impacts of a disaster, and a disaster response, over time. Without these types of data and a more robust evidence base, proactive mitigation of the health effects of public health emergency response on public health workforce and systems will remain limited (Brownson et al. 2018; Calonge et al. 2020). To date, no longitudinal study has been published examining anxiety and depression changes in public health workers specifically. To address this gap, in this study we aim to characterize anxiety and depression in a longitudinal cohort of public health workers in the U.S. and examine changes over time by known risk factors.

## Methods

### Data collection

**Fig. 1:**
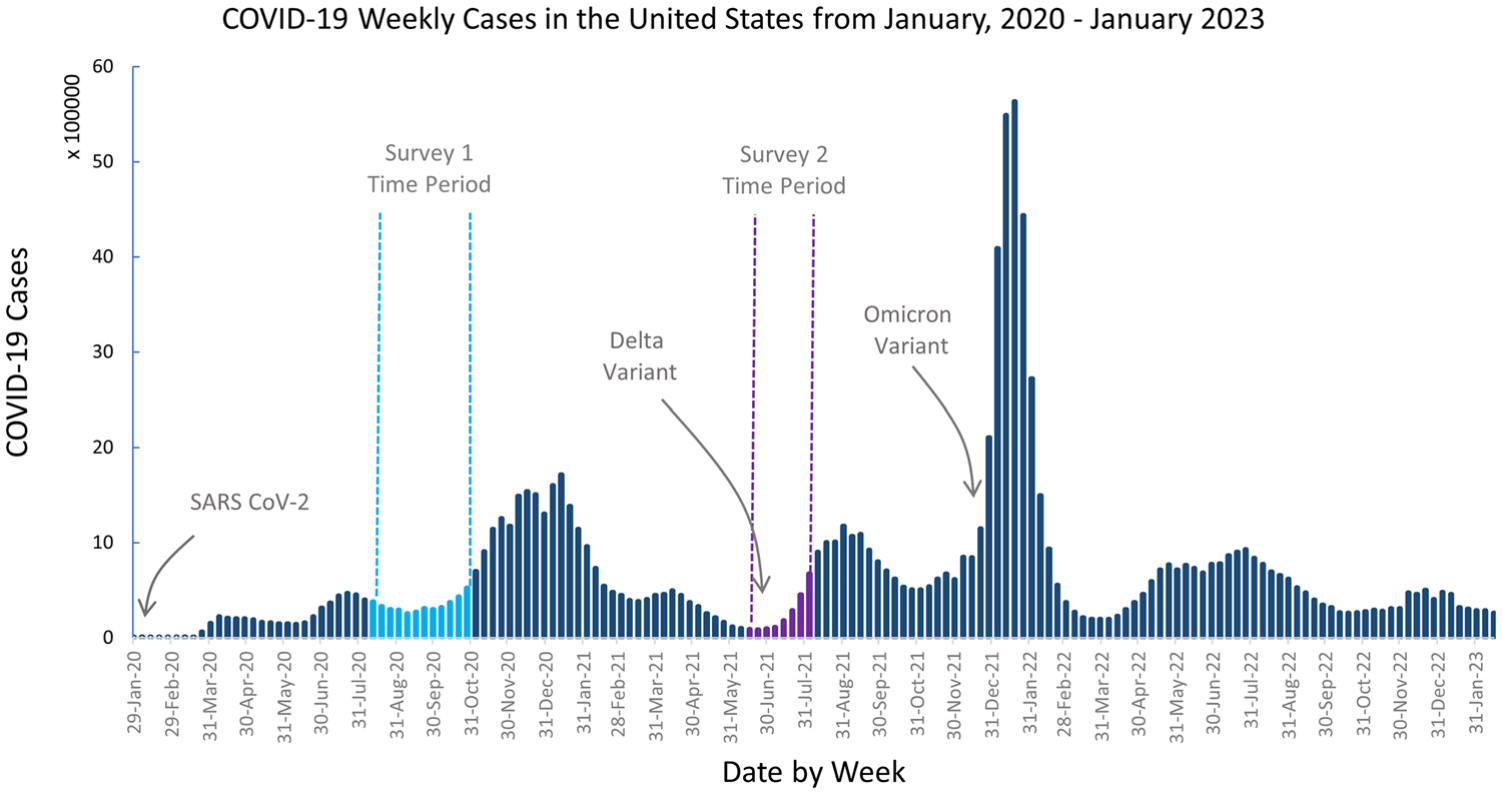
Data collection time periods and COVID-19 weekly case counts in the U.S

Data for this longitudinal study are a sub-sample from a previous cross-sectional study of public health workers in the U.S. (Stone et al. 2021). Inclusion criteria included both working in a governmental public health agency or an academic public health department or program and having work functions that were directly or indirectly impacted by the public health response to the COVID-19 pandemic. Participants indicated on the first survey if they consented to a follow-up survey invitation. Of 390 original respondents, we obtained 85 complete responses in the follow-up survey. Both surveys collected information on demographic characteristics, personal well-being, career plans, and work environment, and both were delivered using the web-based Qualtrics software (Provo, UT). Responses from Survey 1 were collected between August 12 and October 25, 2020, and Survey 2 between June 16 and July 27, 2021 (Fig. 1). Data were cleaned by removing incomplete responses, linking the longitudinal responses by participant name and email, and checking the demographics for consistency between surveys. Survey 2 responses with inconsistent reporting of years of experience in public health were removed.

### Survey measures and questions

Generalized anxiety disorder (GAD) is characterized by excessive and persistent worry or anxiety about everyday life events or activities and is typically accompanied by physical symptoms such as restlessness, irritability, muscle tension, fatigue, and difficulty concentrating (Henning et al. 2007). GAD can significantly impair an individual's daily functioning, affecting their relationships, work, and other important areas of life (Torpy et al. 2011). Depression is characterized by persistent feelings of sadness, hopelessness, and disinterest in activities that were once enjoyable and can also lead to physical symptoms such as fatigue, changes in appetite or sleep patterns, and difficulty concentrating (American Psychiatric Association 2013). Similar to GAD, depression can also increase the risk of other health problems and can interfere with personal relationships and daily functioning (Black et al. 2003; Liu et al. 2020).

We modified the 45-item Survey 1 by adding several questions and scales, forming a 63-item Survey 2. We asked each of the 45-items from Survey 1 again for longitudinal comparison. The first set of questions included sociodemographic characteristics (e.g., age, gender, race/ethnicity, household characteristics, and education) and their professional experience (e.g., years of experience and roles). Questions about personal well-being included respondents' mental health status (e.g., GAD, depression, and burnout). Finally, respondents were asked about career plans and work environment (e.g., hours and days worked).

We used a previously validated 7-item GAD scale (GAD-7) (Spitzer et al. 2006), and the two-item Patient Health Questionnaire (PHQ-2) subscale (Arroll et al. 2010) to assess GAD and depressive disorder. We used a validated, non-proprietary single-item burnout measure to estimate self-reported burnout (Dolan et al. 2015), categorized as some level of burnout (any symptoms of burnout: scale items 3–5) vs. none (scale items 1–2). We assessed social support with two questions from the Social Support Rating Scale (SSRC) that asked about available sources for support for comfort and caring and for economic support and practical problem solving. Sources of support included none, spouse/partner, other family members, friends, relatives, colleagues, companies, official or semi-official organizations such as parties, leagues, and unions, unofficial organizations such as religious organizations or social groups, and other (Xiao 1994; Pfender et al. 2022).

We downloaded data from both surveys from Qualtrics and analyzed them using Stata Version 17 (College Station, TX). We categorized outcomes of interest, i.e., GAD and depression, according to gender, age, race/ethnicity, marital status, household size, work hours, years of experience in public health, public health work sector, and education level. Pearson’s chi-square and Fisher’s Exact were used to determine significant differences at the *p* < 0.05 level for outcomes across various characteristics, sources of support, and work conditions. *T*-tests were used to determine significant differences in overall outcome scoring from Survey 1 and 2. The University of Delaware Institutional Review Board reviewed the surveys and related materials and determined them to be exempt under 45 CFR46.101(b) of the U.S. Department of Health and Human Services regulations for human subjects’ research (IRB# 1641836-1).

## Results

A total of 85 public health workers from 23 U.S. states completed Survey 2 during a 41-day period. Most respondents were currently working in applied public health or governmental public health agencies (72%). The majority were female (81%), White non-Hispanic (75%), under the age of 40 (57%), and had at least a masters-level degree (74%) (Table 1). The proportion of participants that experienced GAD or depression at either time point was categorically similar across each demographic characteristic. This sample was more female than the overall U.S. public health workforce (81% compared to 79% female), more White (75% compared to 54%), and younger (mean age = 40 compared to 47) (de Beaumont Foundation and Association of State and Territorial Health Officials 2022).

**Table 1:** Respondent characteristics by the outcome of generalized anxiety or depression at either time point (*N* = 85)

		Total	None	Anxiety or depression	*p*-Value
		*N* = 85	*N* = 36	*N* = 49	
Gender	Female	69 (81%)	33 (92%)	36 (73%)	0.097
	Male	13 (15%)	3 (8%)	10 (20%)	
	Missing	3 (4%)	0 (0%)	3 (6%)	
Age	18–29	16 (19%)	4 (11%)	12 (24%)	0.47
	30–39	32 (38%)	14 (39%)	18 (37%)	
	40–49	18 (21%)	9 (25%)	9 (18%)	
	50 +	19 (22%)	9 (25%)	10 (20%)	
Race/ethnicity	Non-White	21 (25%)	9 (25%)	12 (24%)	0.96
	White	64 (75%)	27 (75%)	37 (76%)	
Marital status	Single^b^	35 (41%)	12 (33%)	23 (47%)	0.21
	Married/partnered	50 (59%)	24 (67%)	26 (53%)	
Years of experience	1–4 years	20 (24%)	7 (19%)	13 (27%)	0.23
	5–9 years	23 (27%)	7 (19%)	16 (33%)	
	10–14 years	16 (19%)	7 (19%)	9 (18%)	
	15 + years	26 (31%)	15 (42%)	11 (22%)	
Education	≤ Bachelors	11 (13%)	3 (8%)	8 (16%)	0.32
	Masters	52 (61%)	21 (58%)	31 (63%)	
	Doctoral	22 (26%)	12 (33%)	10 (20%)	
Work sector	Applied	61 (72%)	25 (69%)	36 (73%)	0.68
	Other^c^	24 (28%)	11 (31%)	13 (27%)	

COVID-19 = coronavirus disease 2019; Anxiety disorder = respondents who scored ≥ 10 out of 21 on the Generalized Anxiety Disorder (GAD-7) scale; Depressive disorder = respondents who scored ≥ 3 out of 6 on the Patient Health Questionnaire (PHQ-2) scale

^a^Outcomes not reported with less than five respondents

^b^Includes widowed, divorced, separated, never married

^c^Includes academic, clinical setting, non-academic research, nonprofit setting

### Changes in GAD and depression over time

**Fig. 2:**
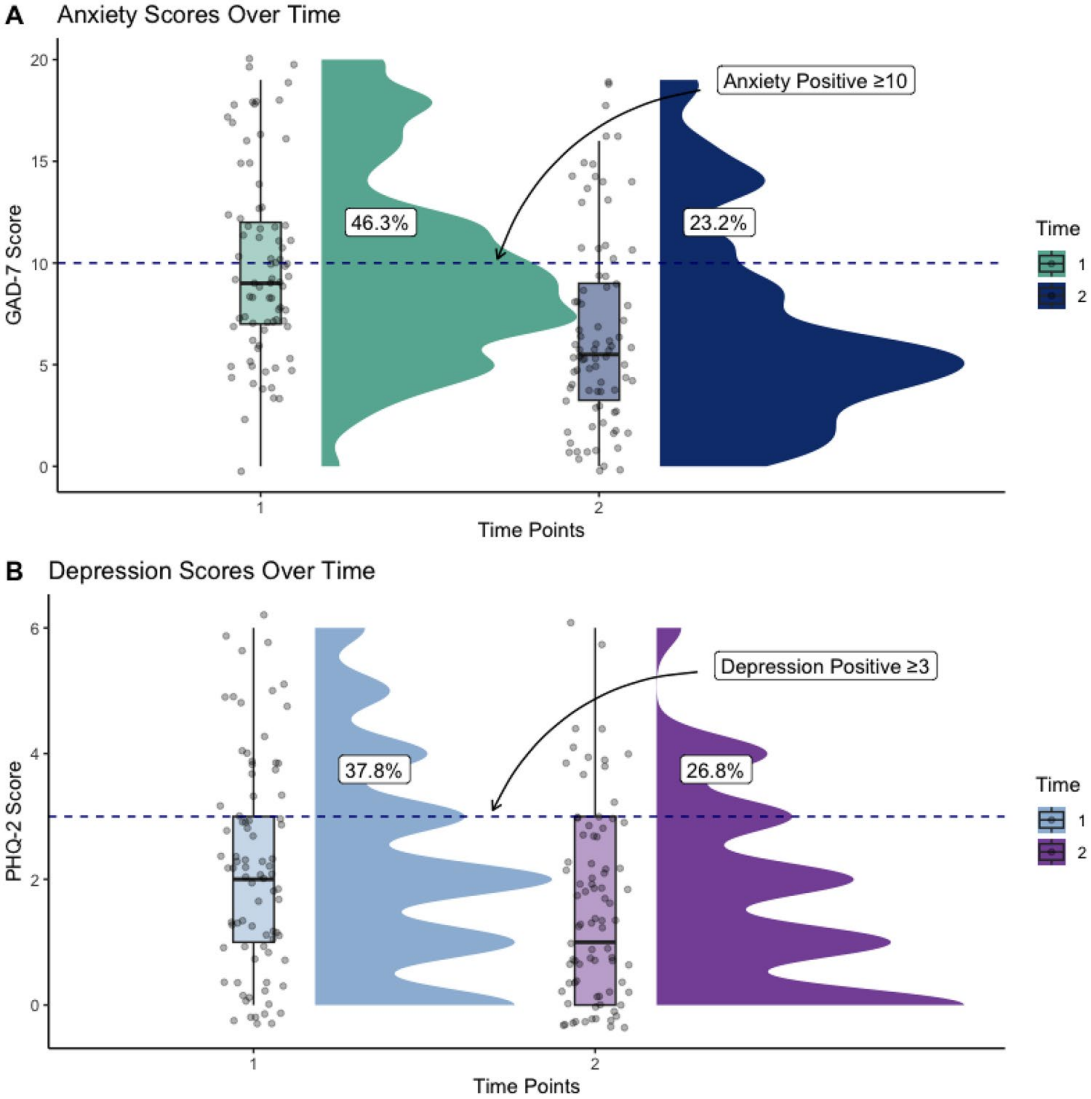
Changes in anxiety and depression scores over time (*N* = 82). **A** Generalized Anxiety Disorder (GAD-7) total score distribution at Survey 1 (green) and 2 (dark blue) with proportion reporting an anxiety score of 10 or more. **B** Depression (PHQ-2) total score distribution at Survey 1 (light blue) and 2 (purple) with proportion reporting a depression score of 3 or more

GAD and depression score totals significantly improved from Survey 1 to Survey 2 (Fig. 2). Anxiety had a greater improvement (mean 1 [M1] = 9.9, 95% confidence interval [CI]1 = 8.8–10.9 compared to M2 = 6.7, CI2 = 5.8–7.9) compared to depression (M1 = 2.2, CI1 = 1.8–2.6 compared to M2 = 1.6, CI2 = 1.3–1.9). The interquartile ranges shifted from Survey 1 to Survey 2 for both GAD and depression. GAD maintained its width in scores (Survey 1 and 2, interquartile range [IQR] = 5) compared to depression (Survey 1, IQR = 2 to Survey 2, IQR = 3), where the range became wider. The proportion of those reporting GAD (46.3% to 23.2%) or depression (37.8% to 26.8%) fell between Survey 1 to Survey 2 overall, with symptoms of anxiety having the largest improvement (Fig. 2).

**Fig. 3:**
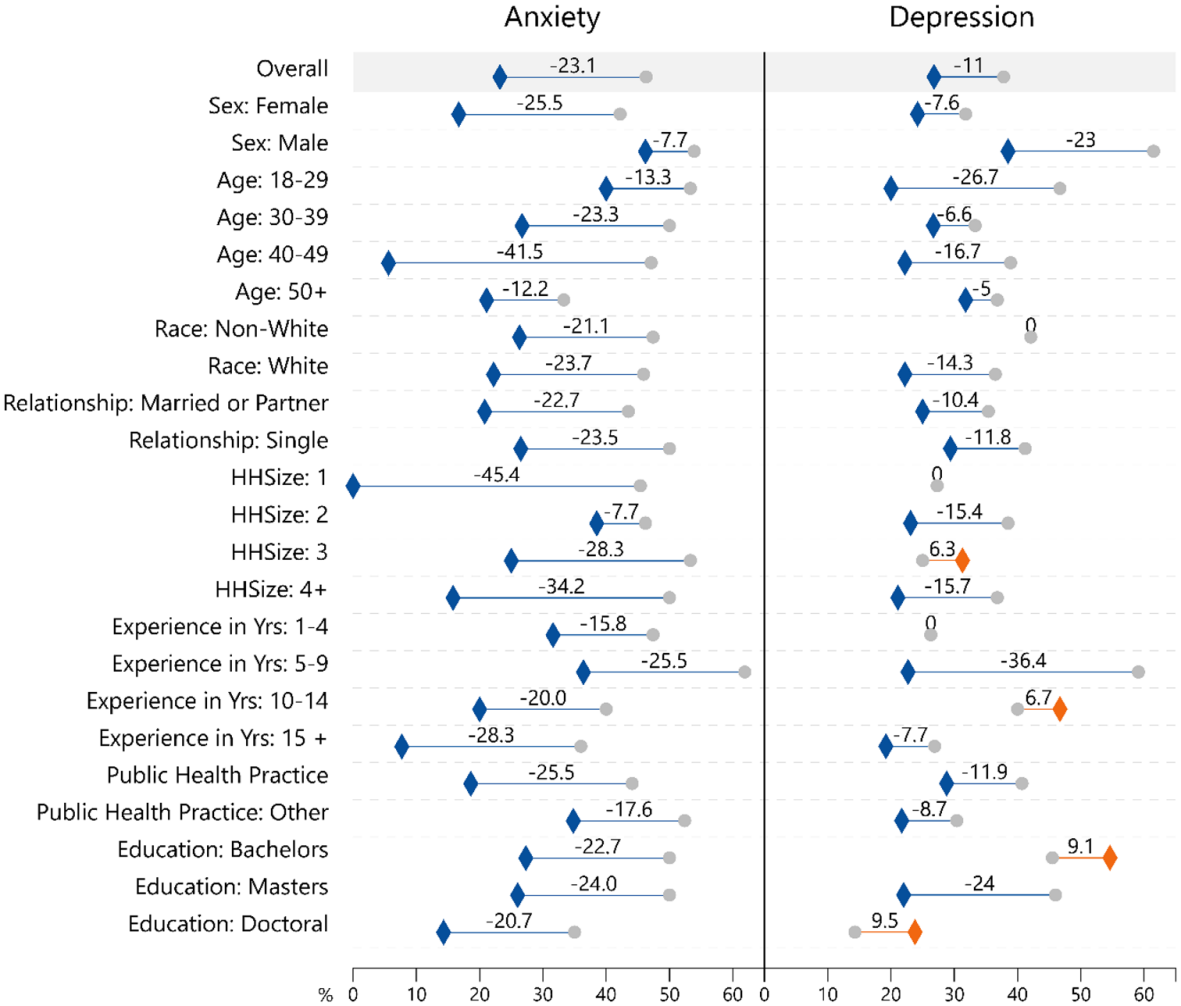
Changes in anxiety disorder and depression disorder prevalence from time 1 to time 2 by respondent characteristics (*N* = 82). Survey 1 = •; Survey 2 = ◊ (orange = worsening; blue = improved); Anxiety = respondents who scored ≥ 10 out of 21 on the Generalized Anxiety Disorder (GAD-7) scale; Depressive disorder = respondents who scored ≥ 3 out of 6 on the Patient Health Questionnaire (PHQ-2) scale

Those that reported symptoms of GAD at Survey 1 saw improvements across all characteristics, notably in females (42.2% to 16.7%), those aged 40–49 (47% to 5%), and those with household sizes of 1 (45.4% to 0%) and 4 + (50.0% to 15.8%) (Fig. 3). Greater improvements in depression were reporting in males compared to females, those with 5–9 years of experience compared to those with more or less experience, and those with a master’s degree compared to those with a bachelor’s or doctorate. However, the proportion of those with depression in some groups increased over time, including those living in a household of 3 (25.0% to 31.3%), those with 10–14 years of experience (40.0–46.7%), and those with a bachelor’s (45.5–54.6%) or doctoral degree (14.3–23.8%). Non-Whites (42.1%), those living by themselves (27.3%), and those with 1–4 years of experience (26.3%) reported no change in depression.

Survey 2 independently investigated the relationship between various work and non-work related conditions and GAD and depression (Table 2). Notably, staying burned out from Survey 1 to Survey 2 was found to be significantly associated with the presence of depression (85.7% vs. 14.3%, *p* = 0.003) at the second time point, but persistent burnout was not significantly associated with GAD. Those that worked 6 or more days per week were more likely to be depressed (72.2% vs. 27.8%, *p* = 0.03). No association was found in hours worked per week and either GAD or depression. The number of social supports considered as comfort and caring or economic and practical changed for most respondents across time, with some experiencing decreases in their number of supports while others experienced increases. Comfort and caring social support change was not associated with GAD or depression. Depression at Survey 2 was significantly associated with the change in economic and practical social support (decreased, 54.6%; stayed the same, 4.6%; increased, 40.9%; *p* = 0.009). Household size and children being present in the home were not found to be associated with either GAD or depression. These findings were consistent with prior risk factors reported by the authors in the original survey populations and elsewhere in the literature (Pfender et al. 2022; Guilaran et al. 2018; Prati and Pietrantoni 2010).

**Table 2:** Generalized anxiety and depression at Survey 2 by work and nonwork-related conditions

	Total	Generalized anxiety	*p*-Value	Depression	*p*-Value
Burnout (*N* = 80)					
Never burned out	17 (21.3%)	3 (16.7%)	0.186	2 (9.5%)	0.003
Stayed burned out	41 (51.3%)	13 (72.2%)		18 (85.7%)	
Became burned out	6 (7.5%)	1 (5.6%)		0 (0%)	
Improved burnout	16 (20%)	1 (5.6%)		1 (4.8%)	
Household size (*N* = 83)					
Stayed the same or decreased	34 (40.9%)	9 (47.4%)	0.794	9 (45%)	0.623
Increased	49 (59.0%)	10 (52.6%)		11(55%)	
Household with kids (*N* = 82)					
No kids ever	26 (31.7%)	6 (31.8%)	0.73	8 (36.4%)	0.618
Now have kids	2 (2.4%)	0 (0%)		1 (4.8%)	
Still have kids (including adding kids)	54 (65.9%)	13 (68.4%)		13 (59.1%)	
No. of social supports—comfort and caring (*N* = 85)					
Decreased	52 (61.2%)	11 (57.9%)	0.828	12 (54.6%)	0.009
Stayed the same	15 (17.7%)	4 (21.1%)		1 (4.6%)	
Increased	18 (21.2%)	4 (21.1%)		9 (40.9%)	
No. of social supports—economic and practical (*N* = 85)					
Decreased	42 (49.4%)	9 (47.4%)	0.912	11 (50.0%)	0.987
Stayed the same	16 (18.8%)	4 (21.1%)		4 (18.9%)	
Increased	27 (31.8%)	6 (31.6%)		7 (31.8%)	
Work days per week (*N* = 70)					
Less than 6	35 (50%)	6 (42.7%)	0.55	5 (27.8%)	0.029
6 or more	35 (50%)	8 (57.3%)		13 (72.2%)	
Hours per week (*N* = 79)					
Less than 50	52 (65.8%)	11 (64.7%)	0.913	12 (57.1%)	0.328
50 or more	27 (34.2%)	6 (35.3%)		9 (42.9%)	

Generalized Anxiety = respondents who scored ≥ 10 out of 21 on the Generalized Anxiety Disorder (GAD-7) scale; Depression = respondents who scored ≥ 3 out of 6 on the Patient Health Questionnaire (PHQ-2) scale; *p*-values = from Pearson's χ^2^ or Fisher's Exact in each subcategory

## Discussion

This longitudinal study of 85 public health workers found that GAD and depression scores significantly improved between 2020 and 2021. Those that reported symptoms of GAD at Survey 1 saw improvements across all demographic groups, while those with depression at Survey 1 showed worsening in some strata.

GAD is a condition of excessive worry about everyday issues and situations that can last longer than 6 months (National Institute of Mental Health, n.d.). The duration of an anxiety disorder can vary from person to person. Several longitudinal studies have assessed the impact of the COVID-19 response on groups including college students, medical students, and healthcare and other workers (Baumann et al. 2021; Hines et al. 2021; Hu et al. 2020; Li et al. 2020; Saraswathi et al. 2020; Van Steenkiste et al. 2022). In one study of the general population of the Netherlands, among those with an anxiety disorder, the median episode duration was 7.5 months and the mean duration was 15.2 months; 38.8% had not recovered at 12 months and 30.1% had not at 36 months (Ten Have et al. 2021). A 1-year longitudinal study conducted in Singapore investigated changes in depression and anxiety among frontline emergency department healthcare workers during the COVID-19 pandemic and found a significant improvement in anxiety among all healthcare workers, while depression worsened among doctors (Th’ng et al. 2021). Another study found that anxiety significantly increased over time among healthcare workers, with the primary driver of increased anxiety over time being the threat of COVID-19 contagion risk (López Steinmetz et al. 2021).

GAD and depression were high among multiple groups after the full extent of the early pandemic was realized; this is reflected in the findings at initial survey. Comparing self-reported mental health among college students prior to the start of the implementation of control measures in Wuhan, China, and 2 weeks after the start of a strict lockdown, Li et al. (2020) reported that fear of infection and a lack of access to adequate supplies increased the potential for negative psychological consequences. Among undergraduate medical students in India, a longitudinal assessment of depression, anxiety, and stress showed increases in both prevalence of these conditions as well as the severity of both anxiety and stress between December 2019 and June 2020 (Saraswathi et al. 2020). Among medical students in China, few reported that the COVID-19 pandemic had a negative impact on their career choices; however, this study compared data collected in November 2019 and February 2020, which was still quite early in the trajectory of the pandemic and thus may not have fully captured longer-term adverse psychological or occupational impacts of the pandemic (Hu et al. 2020).

In our study, the time between data collection ranged from 8–11 months. The first survey was during summer of the COVID-19 pandemic response, some 6–8 months since the beginning of the pandemic when no vaccine was available. The second survey was during summer 2021, when vaccines were widely available. Vaccine availability has been shown to decrease anxiety in health workers. For example, people who were vaccinated between December 2020 and March 2021 reported decreased mental distress levels in surveys conducted after receiving the first dose (Perez-Arce et al. 2021). Another study found a 30% reduction in anxiety and depression in the general public after receiving at least 1 dose of the vaccine in May 2021 due to alleviated fears of being infected with SARS-CoV-2 (Agrawal and Imondi 2022). Similarly, a U.S. Census Bureau Household Pulse Survey showed overall decreases in anxiety and depression from August to December 2020 to December 2020 to June 2021 (Jia et al. 2021). Conversely, a large population-based study by Ettman et al. (2022) reported persistent and increasing depressive symptoms from March 2020 to March 2021 (Ettman et al. 2022). In our study, generally, depressive symptoms subsided from Survey 1 to Survey 2. However, several characteristics and experiences were related to increases in depressive symptoms, like having a bachelor’s degree or doctoral degree, having a household size of 3, and having work experience between 10–14 years. Identifying as non-White, living alone, or having only 1–4 years of experience indicated persistent depression.

Burnout is a state of mental and emotional exhaustion that can result from prolonged stress or frustration. It is often accompanied by feelings of cynicism and a lack of personal achievement. While burnout shares some similarities with depression, such as a loss of interest and difficulty concentrating, there is an ongoing debate among researchers about whether the two conditions are distinct or overlapping (Bianchi et al. 2015; Koutsimani et al. 2019). A meta-analysis in 2019 examined the relationship between burnout and depression and burnout and anxiety, finding there was no conclusive overlap between them, showing them to be distinctly different (Koutsimani et al. 2019). In our study, those that stayed burned-out over time were more likely to have depressive symptoms, but this relationship was not consistent for anxiety. This is reflective of studies with both patient-facing and public health workers and time spent working (Pfender et al. 2022; Lang et al. 2020; Lee and Park 2022; Saade et al. 2022). However, our findings did not show a significant association between hours worked and anxiety or depression over time, only in days per week with depression.

Social emotional support, such as having someone to confide in, has consistently been reported as a protective factor against depression in adults that can directly reduce negative emotions associated with distressing situations (Gariépy et al. 2016). Emotional support may be more likely to be utilized in situations where there is a greater need, and thus could be experienced differently over time depending on someone’s level of distress (Seeman 1996). A significant association between changes in the number of social supports for comfort and caring (emotional) and depressive symptoms at Survey 2 was observed in our survey. Those that indicated no change in the number of supports were less likely to have depression at Survey 2. Over the course of the pandemic, professional public health associations and the media documented ongoing and increasingly severe harassment of, and threats to, public health workers, particularly public health leaders, which may have made social supports less available or more difficult to access. Assuring ongoing access to supports, along with additional training and coping support, will be critical as part of emergency preparedness planning for public health workers going forward. Social supports available in the workplace such as increased access to counselors, as well as more time to utilize those services as less uncertainty about the transmission of COVID-19 with the introduction of vaccines, may be related to improvement in depression.

This study has several important limitations. The recruitment of participants for the follow-up study, which involved individuals who had participated in the initial survey, was a challenge as many public health workers changed jobs or left the public health workforce entirely during the first year of the pandemic response. As a result, the response rate for Survey 2 was only 21.7%, which could have introduced selection bias. The demographics of those responding to Survey 2 were not significantly different from Survey 1. Comparing follow-up participants to those that only completed Survey 1, we found no significant difference in gender, age, race/ethnicity, marital status, years of experience, education, and by work sector and thus assumed these were comparable populations observed under different exposure conditions. However, other potential confounders were unmeasured in the survey and the survey was closed earlier than planned due to increased workloads associated with the Delta variant wave**.** The sample for this longitudinal study was also more female, Whiter, and younger than estimates of the overall U.S. public health workforce, and thus the findings are not generalizable. However, the prevalence of mental health disorders reported here among public health professionals who participated in the COVID-19 response is concerning, and additional studies of more well-defined or representative occupational groups are needed. Unlike much of the other published literature, which focused on assessing changes over a period of only a few weeks to a few months of the COVID-19 pandemic response, this study collected responses over the first year including the time during which the COVID-19 vaccine was made widely available and across the U.S. as well as during different surges. Other known longitudinal studies were all short term (less than 3 months) and were a convenience sample.

## Conclusion

Public health workers experienced elevated levels of anxiety and depression in the initial year of the pandemic response, but the prevalence of these symptoms was reduced by the summer of 2021 after vaccines had become widely available. However, persistent depression was associated with burnout, working more than 50 h per week, and the availability of emotional support. To some extent, these risk factors could be mitigated through training, planning, and building administrative capacity prior to the next disaster or public health emergency response. While there were reductions in the prevalence of GAD, improvements in depression were observed only in specific groups. These findings highlight the importance of targeted and sustained mental health supports in public health workplaces that incorporate strategies for addressing burnout, difficult work schedules, and access to emotional support.

## Data Availability

The datasets generated during and/or analysed during the current study are available from the corresponding author on reasonable request.
